# Polyhedra structures and the evolution of the insect viruses

**DOI:** 10.1016/j.jsb.2015.08.009

**Published:** 2015-10

**Authors:** Xiaoyun Ji, Danny Axford, Robin Owen, Gwyndaf Evans, Helen M. Ginn, Geoff Sutton, David I. Stuart

**Affiliations:** a Division of Structural Biology, The Wellcome Trust Centre for Human Genetics, University of Oxford, Roosevelt Drive, Oxford, Oxfordshire OX3 7BN, United Kingdom; b Diamond House, Diamond Light Source, Harwell Science & Innovation Campus, Didcot, Oxfordshire OX11 0DE, United Kingdom

**Keywords:** SeMet, selenomethionine, Protein microcrystals, Polyhedra, *In vivo* crystals, Cypovirus, Micro focus crystallography

## Abstract

Polyhedra represent an ancient system used by a number of insect viruses to protect virions during long periods of environmental exposure. We present high resolution crystal structures of polyhedra for seven previously uncharacterised types of cypoviruses, four using *ab initio* selenomethionine phasing (two of these required over 100 selenomethionine crystals each). Approximately 80% of residues are structurally equivalent between all polyhedrins (pairwise rmsd ⩽1.5 Å), whilst pairwise sequence identities, based on structural alignment, are as little as 12%. These structures illustrate the effect of 400 million years of evolution on a system where the crystal lattice is the functionally conserved feature in the face of massive sequence variability. The conservation of crystal contacts is maintained across most of the molecular surface, except for a dispensable virus recognition domain. By spreading the contacts over so much of the protein surface the lattice remains robust in the face of many individual changes. Overall these unusual structural constraints seem to have skewed the molecule’s evolution so that surface residues are almost as conserved as the internal residues.

## 1 Introduction

Cypoviruses, members of the family *Reoviridae*, subfamily *Spinareovirus*, possess a single capsid layer with turrets and are commonly embedded in crystalline occlusion bodies called polyhedra, which are formed in the cell cytoplasm and mainly composed of a single virus-encoded protein, polyhedrin (Attoui et al., 2012). Cypoviruses, which generally possess 10 double-stranded RNA genome segments (polyhedrin is encoded by the smallest), are important insect pathogens (Rothman and Myers, 1996), having been isolated from a wide range of insects – Lepidoptera (80%), Diptera (16%), Hymenoptera (3%), Coleoptera and Neuroptera (<1%) (Belloncik and Mori, 1998).

Cypoviruses have been classified into 21 distinct types (Attoui et al., 2012). Within a type the amino acid sequence of polyhedrins are highly conserved, whilst between types there is little conservation. The structure of Bombyx mori cypovirus 1 (CPV1) polyhedrin, determined by micro-focus crystallography, showed the polyhedra to be built from rigid trimeric building blocks, which assemble with the aid of ribonucleoside triphosphates (NTPs) to form a lattice containing very little bulk solvent (Coulibaly et al., 2007). To ensure the delivery of virus particles to the target intestinal cells, allowing the transmission of packages of infectious virus between hosts by oral-faecal routes, polyhedra are disrupted only when they are exposed to the very alkaline pH environment found in insect midguts (Vago and Croissant, 1959). Region-specific ion transport has been found to facilitate the alkalinisation of the anterior midgut lumen with major roles for an H^+^ V-ATPase-energised Cl^−^/HCO_3_^−^ exchanger and carbonic anhydrase (Boudko et al., 2001), consistent with carbonate ions being used to modulate the pH of the midgut.

We recently reported polyhedrin structures for CPV18, a close relative of CPV1, determined using synchrotron radiation from undisrupted insect cells and from purified polyhedra (Axford et al., 2014), and for CPV17 at room temperature and at 100 K, using an X-ray free electron laser and synchrotron respectively (Ginn et al., 2015b). Here we describe polyhedrin structures for all other types of cypoviruses for which polyhedra could be obtained, giving us a database of nine structures from which we derive rules for crystal formation and dissolution and investigate how preservation of this unusual phenotype (the crystal lattice) has impacted on the variation of the protein over geological time scales.

## 2 Material and methods

For readability we refer to the cypovirus type and strain by number alone i.e. CPV14 for Lymantria dispar cypovirus 14, as defined in Table S1. For completeness we include CPV1 (Coulibaly et al., 2007) as a reference in our analyses.

### 2.1 Expression and purification of polyhedra

The cypovirus types studied in this paper are abbreviated as detailed in Supplementary Table S1. cDNA pools of cypovirus genomes for types CPV1, CPV5, CPV14 and CPV15 were a gift from Dr. Shujing Rao (Institute for Animal Health, Pirbright, Surrey, UK). Polyhedrin genes of CPV4, CPV17, CPV18, CPV19 and CPV20 were synthesized by GeneArt (Life Technologies) using sequences obtained from GenBank.

*Spodoptera frugiperda* (*Sf*9) cells were passaged at 27 °C in Sf-900 II serum-free medium (SFM, Life Technologies) using standard procedures (O’Reilly et al., 1994). The coding regions of genome segment 10 from CPV1, CPV5, CPV14 and CPV15 were amplified and inserted in the multi-cloning site of vector pFastBac1 (Life Technologies). The recombinant plasmids were transformed into competent DH10Bac cells (Life Technologies) to produce bacmid DNA for the generation of recombinant baculoviruses, following the protocol of the Bac-to-Bac® baculovirus expression system (Life Technologies). The polyhedrin genes from CPV4, CPV17, CPV18, CPV19 and CPV20 were sub-cloned into the transfer vector pBacPAK9 (Clontech). Base domain deletion mutants of CPV1 (CPV1Δ) and CPV4 (CPV4Δ) were produced by three steps of PCR using primers encoding a Gly-Ser-Gly linker (described in (Strachan and Read, 1999)). The recombinant virus was produced by co-transfection of linearized baculovirus DNA and the transfer vector following a standard protocol (Zhao et al., 2003). Polyhedra were expressed and purified as described earlier (Anduleit et al., 2005). SeMet-labelled polyhedra were generated as published previously (Ji et al., 2010) and the incorporation of SeMet was confirmed to be at least 80% using mass spectroscopy. Purified polyhedra were observed and characterised by a Zeiss Axiovert 200 M inverted microscope with fluorescence/phase or DIC (Nomarski) imaging.

### 2.2 Data collection

Polyhedra were spread on a MicroMesh mount (Mitegen, Ithaca, USA) in a solution of 10 mM HEPES, pH 7.5 and 50% ethylene glycol and flash-frozen in a stream of nitrogen gas at 100 K. X-ray datasets were collected at beamline I24 (Diamond) on either a Rayonix MX300 (Rayonix, Evanston, USA), a PILATUS 6 M or a PILATUS3 6 M detector (DECTRIS, Baden, Switzerland). The typical 10 μm × 10 μm focal spot at the sample position of beamline I24 was reduced with the use of beam defining slits; in the case of CPV17, slits close to sample position and producing a beam size of ∼4 μm × ∼4 μm with ∼1 × 10^11^ Ph/s; in the case of all other CPVs, slits at the focal point of the first of two pairs of KB mirrors that form the optical configuration of the beamline, producing a beam size of ∼6 μm × ∼6 μm with ∼1.5 × 10^11^ Ph/s (Evans et al., 2011). Data were processed using various programs including Denzo/Scalepack (Otwinowski et al., 1997), XDS (Kabsch, 1993), Mosflm/Scala (Leslie and Powell, 2007) and XIA2 (Winter, 2010) according to the detector types. The current in the storage ring was maintained around 200–250 mA at Diamond. Each crystal was exposed to 100% transmission of the X-ray beam with a fine sliced oscillation range of 0.05–0.1° for best performance of the PILATUS 6 M detectors. For the smallest crystal only 10–20 frames could be collected. Data from a number of crystals of each cypovirus including native and SeMet-substituted crystals were merged respectively to give a series of datasets with adequate redundancy (Table 1). Details for the data collection strategy of CPV17 at the Linac Coherent Light Source X-FEL (Stanford, U.S.A.) are described in (Ginn et al., 2015a,b).

### 2.3 Structure determination and refinement

The structure of CPV5 polyhedra was solved using single isomorphous replacement with anomalous scattering (SIRAS). Seven heavy atom sites were found and position refined at 3 Å by SHARP (Fortelle and Bricogne, 1997). SHARP figures of merit were (centric/acentric) 0.37/0.44 (36.0–3.0 Å) and 0.19/0.28 (3.1–3.0 Å). Phasing power (isomorphous/anomalous) was 1.202/0.897. Solvent flattened electron density maps from SOLOMON (Abrahams and Leslie, 1996) and DM (Cowtan, 1994) at 3 Å resolution were used for the initial model building using Coot (Emsley and Cowtan, 2004). Iterative model building and refinement were performed by Coot and BUSTER (Bricogne et al., 2011). Final refinement used PHENIX (Adams et al., 2010) at a resolution of 2.07 Å.

The structures of other cypovirus polyhedrins were solved using a method which combined molecular replacement and SIRAS. The molecular replacement was performed using PHASER (McCoy et al., 2007) with the mainchain atoms of known cypovirus polyhedrin structures as search models against the native data. As we solved additional polyhedrins more starting models could be used for molecular replacement. We also optimised the process by selecting those cypovirus types which bore the highest sequence similarity. The resulting phases were used to calculate anomalous and isomorphous difference Fourier maps using FFT (Read and Schierbeek, 1988; Winn et al., 2011). Potential heavy atom sites were found and positions refined by SHARP (Bricogne et al., 2003) with the phases from molecular replacement not only as a restraint but also helping the refinement. The phases from SIRAS and molecular replacement were finally combined to be the restraint phases in BUSTER (Bricogne et al., 2011) during the process of refinement. Model building was performed in Coot (Emsley and Cowtan, 2004) and final refinement used PHENIX (Adams et al., 2010). All the structures were validated by MolProbity (Chen et al., 2010). Details of the refinement are in Table 2.

Molecule interfaces and oligomerization were evaluated by PISA server at the European Bioinformatics Institute (Krissinel and Henrick, 2007). Three-dimensional structure alignment was performed and analysed by SHP (Stuart et al., 1979). Illustrations were generated by Pymol (Schrodinger, 2010).

### 2.4 Alkaline disruption

Initially each of eleven polyhedra samples (CPV1, 4, 5, 14, 15, 17, 18, 19, 20, 1Δ and 4Δ) were suspended in 500 μl alkaline buffer (50 mM NaHCO_3_, pH 10.5) at room temperature. The optical density of the sample was measured at regular time intervals by a spectrophotometer at the wavelength of 600 nm (OD600). The measurements were compared with the starting value to characterize the ratio of dissolved protein to total. In a follow-up experiment aliquots (20 μl) of CPV1, 4, 1Δ and 4Δ polyhedra suspended in water were taken and centrifuged at 20,000×*g* for 1 min. The pellet was dissolved in 50 μl buffer solution (50 mM) at room temperature for 20 min. Five different alkaline buffers were used: Carbonate pH 10.5, Carbonate pH 10, CAPS (N-cyclohexyl-3-aminopropanesulfonic acid) pH 10, Glycine pH10 and Borate pH 10. The sample was centrifuged again to separate the soluble and insoluble material. The quantities of material dissolved in the alkaline buffers were measured by the absorbance at 280 nm using a ND-1000 Spectrophotometer (NanoDrop).

## 3 Results

### 3.1 Structure determination

The polyhedrin genes from eight cypovirus types (4, 5, 14, 15, 17, 18, 19 and 20) were cloned and recombinant baculoviruses generated (Material and Methods). The resultant recombinant cypovirus polyhedra varied in size from 6 to 8 μm for CPV4, to ∼1 μm for CPV17 (Table 1). The use of a small (∼5 μm × 5 μm) beam at the tuneable micro-focus I24 beamline at Diamond Light Source allowed data collection at 100 K and structure solution at 2.2 Å resolution or beyond for all of these polyhedra (except that the initial structure determination of CPV17 required the use of an X-FEL see below, Table 1). Due to the tiny size of the crystals, data from a number of crystals were merged to attain a complete dataset with sufficient signal to noise. In four cases the structures were solved by SeMet labelling, and for two of these over 100 crystals were required to achieve sufficient anomalous signal to solve the structure (Table 1). The final structures were all reliable (Table 2). Data for CPV17 were also collected at room temperature on the CXI beamline at the Linac Coherent Light Source, to a resolution higher than could be achieved on I24. The small differences between the CPV17 100 K and room temperature structures have been described (Ginn et al., 2015b). For CPV1 the resolution was 2.1Å with R_free_ 12.9% and R_work_ 19.6% (Coulibaly et al., 2007).

### 3.2 The fold of the core structure of polyhedrin is highly conserved

**Fig. 1:**
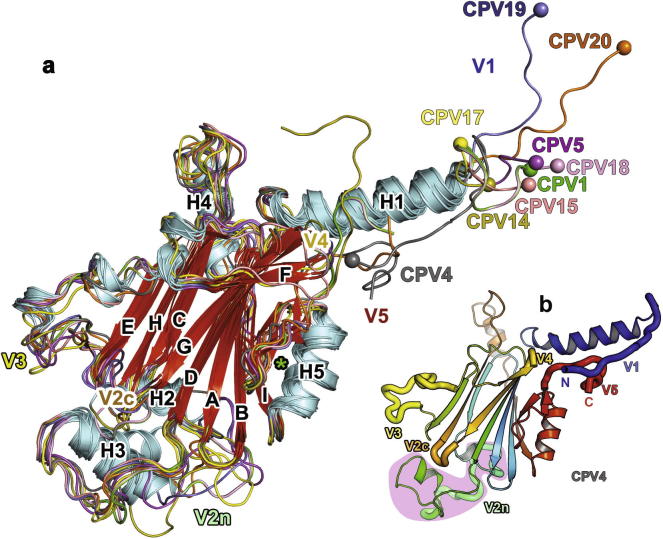
Overall structure. (a) Cartoon representation of the structures of the polyhedrin subunit for 9 cypovirus types. Structures were aligned using SHP (Stuart et al., 1979). Secondary structure elements are marked and coloured cyan for helices and red for β-sheet with the loops coloured by cypovirus type; CPV1, green; CPV4, grey; CPV5, magenta; CPV14, olive; CPV15, salmon; CPV17 yellow; CPV18, pink; CPV19, slate; CPV20, orange. N-termini are highlighted by a sphere. The variable regions are labelled V1–V5. (b) Cartoon representation of the CPV4 polyhedrin subunit coloured from blue to red, N-terminus to C-terminus. Variable regions are highlighted by thickened “worms” and are labelled. The green star by H5 marks the conserved interaction shown in Fig. 3c.

All polyhedra belong to the same *I*23 space group and the native crystals (at 100 K) mostly possess a unit cell of 102–103 Å (102.8 ± 0.4 Å). The exceptions were CPV4, which forms crystals with 2 distinct unit cell dimensions (101.7 and 104.8 Å – the structure of the larger unit cell is an outlier and is discussed below), and CPV17 which has larger cell dimensions (106.1 Å room temperature and 104.9 Å 100 K). The fold of all the polyhedrins is highly similar – a β-barrel core sitting on a base domain, surrounded by a common array of five helices (H1–H5, Fig. 1a). Fig. 1b shows the arrangement of the major secondary structure elements along the polypeptide chain. The β-barrel core is the most conserved part of the structure – 90% of Cαs align with rmsd 1.1 Å (Fig. S1) and non-aligned residues lie at the periphery of the core and the termini. The β-core is reminiscent of the jelly-roll fold seen in the capsids of many icosahedral viruses (Koonin, 2008) (Fig. 1a), however, the arrangement of the nine β-strands in the two β-sheets, IBADGF and CHE, is novel. The base domain lies between H2 and βD, comprises H3 and the loops connecting it to the core and varies in length from 33–41 residues between the different cypovirus types (Fig. S2). The base caps an exposed side of the β-sheets and interacts with the loops connecting the β strands. Despite little amino acid sequence identity (Fig. S2) the 3-D structures of the polyhedrin subunits can be superimposed on one another pairwise such that 203 Cαs (∼80%) are aligned with mean rmsd 1.5 Å. The full length polyhedrin structures are generally well ordered, as expected for a crystal lattice which has extensive interactions and low solvent content (19–25%), however some types show regions of flexibility: 13 residues (136–148) of CPV5 and 6 residues (68–73) of CPV14 have high (>40 compared with an overall Wilson B-factor of 21.3) B-factors, whilst 13 residues (66–78) of CPV15 could not be modelled (Fig. S2). These areas are in structurally variable parts of the proteins.

Comparison of the different polyhedrins reveals five variable regions: the N-terminal loop, connections between secondary structures (H2 and H3, βE and βF, βF and βG, βG and βH), and the C-terminal loop, which we designate V1–V5 respectively (Fig. 1). V2 forms a ‘cap’ at one end of the protein and is subdivided across two sections of the polypeptide, V2n and V2c (Ginn et al., 2015b). Differences in these regions give each polyhedrin its characteristic appearance. CPV4 features long V1 and V5, which loop back to interact. CPV5 has insertions at V3 and V2c which form two hairpins resembling a clamp. CPV14 essentially lacks a V1 extension whereas a long V5 loop forms a hook and an intra-molecular disulphide bridge helps to stabilise the base domain. CPV15 is like CPV5, but with a smaller clamp, and has a dramatic conformational change in V4. CPV17 is unique in having an incompletely (∼50%) formed inter-chain disulphide bond at room temperature, which may confer mechanical stability (Ginn et al., 2015b). Baculovirus polyhedra also contain a disulphide bond, involving a single cysteine, which links to form a dodecamer (Ji et al., 2010). As expected from the 83% amino acid sequence identity, CPV18 looks very similar to CPV1. CPV19 possesses a combination of a long V1 and V2c hairpin. CPV20 has elongated V1 and V5 but unlike CPV4 they do not interact. The V5 regions of CPV5, 15, 17 and 19 differ, but all end in a similar place. Despite a difference in unit cell of only ∼0.2 Å, native and SeMet crystals of CPV5 show a conformational change: in SeMet crystals pairs of loops 166–169 and 135–143 protrude to meet and coordinate a Ca^2+^ with Asp138 and Asp168 (Fig. S3), whilst in native CPV5 there is no evidence for the Ca^2+^, electron density is poor for 136–148, and the 135–143 loop shifts, possibly due to the proximity of methionine/SeMet residues at 136 and 170. There is no obvious significance to this change.

When we finally solved all of the structures of these polyhedra it became clear that they are all closely structurally related. This is remarkable since, although they are not totally novel structures, the low sequence identity, regions of structural variability and extremely low solvent content, rendered molecular replacement was almost impossible.

### 3.3 Very few amino acids are conserved

Only two residues are conserved across the nine structurally aligned polyhedrins. When CPV17 is excluded this increases to five (highlighted in Fig. S2: Q17, G95, P106, E217 and Y232, using CPV1 numbering), all located in non-variable regions. Q17 lies in H1 and the side chain engages the main chain of a distal part of the molecule – NE2 with the carbonyl group of residue 149, and OE1 with the amino group of residue 165. E217 and Y232 are towards the C-terminal region, outside the core. E217 OE2 forms a hydrogen bond with Y232 OH, and an ionic interaction with R13 in a symmetry related molecule, except in CPV5 and CPV20. E217 OE1 forms a hydrogen bond with the amino group of residue 235. P106 bridges the base domain and strand βD, and its limited flexibility likely helps direct the correct domain organisation. G95 is in the base domain close to H3, indeed only a glycine residue would allow the required packing of H3.

### 3.4 Crystal contacts and assembly are functionally conserved features

**Fig. 2:**
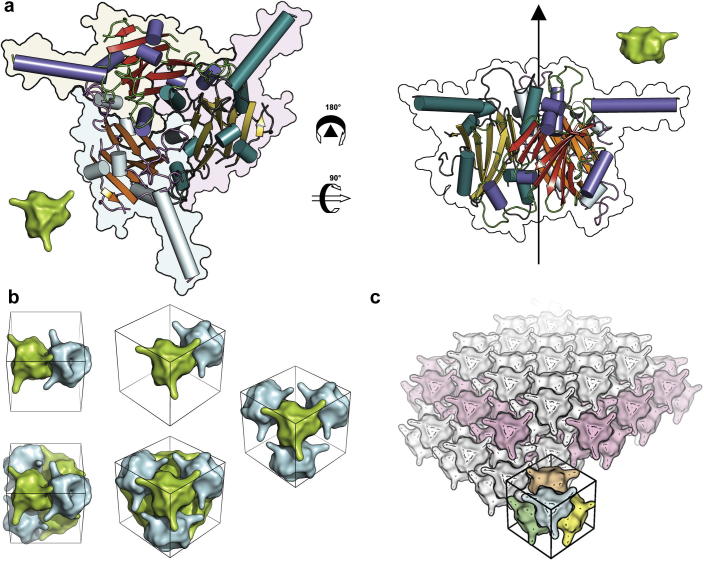
From trimer to crystal. (a) Two views of a cypovirus trimer coloured by secondary structure. In the left panel (viewing the trimer from the ‘top’) each polyhedrin monomer is delineated by a different background colour. The arrow on the right panel goes through the threefold axis. The smaller green trimers are for orientation aids for panel (b). (b) Trimers can come together progressively. Trimers facing ‘inwards’ and ‘outwards’ have been coloured cyan and green respectively to aid interpretation and show the view. The unit cell is marked for clarity although it is unlikely that assembly will occur solely within it. (c) Multiple copies of the dodecamer are arrayed in the crystal. For one dodecamer each trimer is coloured individually. For clarity the dodecamers at the 1/2, 1/2, 1/2 position are coloured light pink. For all the panels the ‘core’ structure shown is that of CPV5 using just the residues which are aligned between all the 9 polyhedrin structures. Structural alignments were performed with SHP (Stuart et al., 1979). The surface maps have been progressively smoothed which results in a slightly coarse and stylised rendering but which helps to visualise the complicated interactions.

To investigate whether the conservation of the molecular structure and crystal packing arises from conserved interactions, the inter- and intramolecular interactions made by residues were plotted against residue number (Fig. S4). This confirms that both the inter- and intramolecular interactions are broadly conserved across all nine polyhedra. The strongest intermolecular interactions define a trimer composed of a cluster of three β-barrels sandwiched by helices top and bottom (Fig. 2a). Strong hydrophobic interactions and large numbers of hydrogen bonds, mainly involving H3, H4, elements within V3 and sheet-CHE, bind the trimer around the body diagonal threefold axes. Some 38% of the amino acids in each subunit participate in the interface, which buries ∼3500 Å^2^ of surface area (Fig. S4a). Three outstretched arms, formed mainly by H1 mediate, in part, a complex higher-level assembly, with each trimer contacting eight others. These arms stretch to contact another trimer on the far side of the unit cell (translated in the direction of a cell edge). The strongest trimer-trimer interactions are also lateral, involving trimers tightly packed along a cell edge, forming contacts of ∼8000 Å^2^ per trimer (involving ∼55% of amino acids, Figs. 2b and S4b). A further interaction of ∼28% of the polyhedrin amino acids, burying ∼1500 Å^2^ of surface per trimer (Figs. 2b and S4b) is also formed. The net effect of these lateral interactions is to form layers of molecules in all three directions, stacked alternately (Fig. 2c). As the network of trimers builds up, four trimers are brought close together around (0, 0, 0) and (1/2, 1/2, 1/2), to form a dodecameric arrangement and there is interaction mainly between the base domains (Figs. 2b and S4b) (involving around 9% of amino acids, and burying ∼900–1800 Å^2^ per trimer). The sum of such interactions, from which the whole network of a polyhedron crystal can be constructed, involves the majority of the surface of the trimer (Fig. S4b).

Overall there is remarkable strength and redundancy in the interactions, with no single weak point, and ambiguity in the assembly pathway may be key to the molecule’s function, flexibility in assembly allowing the lattice to remain intact whilst building around virus particles.

### 3.5 The base domain implicated in viral recognition is dispensable

Native crystals of CPV4 polyhedra grouped into two distinct unit cell dimensions, 101.7 (±0.27) Å and 104.8 (±0.18) Å, whilst all SeMet crystals had the larger size (Table 1). Crystals with both unit cell dimensions were observed on each of several frozen meshes suggesting that this was not an artefact of sample preparation. In the larger-cell structure, residues 1–12, 75–108, 189–192 and 254 were not visible, however SDS–PAGE analysis of crystals showed only a single protein band, corresponding to the full length polyhedrin, suggesting that this is not due to cleavage. Residues 75–108 and 189–192 are clustered around the centre of the dodecameric assembly described above. In the absence of these trimer–trimer interactions the trimers of the dodecamer slide away from teach other, enlarging the unit cell. The volume of the central cavity increases from 7220 Å^3^ to 18,784 Å^3^, and the solvent plus disordered protein fraction of the crystal becomes 41% (compared to a solvent content of 19% in the smaller cell crystals). Since residues 75–108 form the base domain we wondered if polyhedra crystals could be formed without the base domain. To test this, polyhedrin proteins were made with the base domain removed by deleting residues 74–110 of CPV4 and 71–103 of CPV1 and replacing them with a flexible Gly-Ser-Gly linker (resulting in CPV4Δ and CPV1Δ respectively). Both formed diffraction capable crystals (Table 1). The CPV4Δ structure was very similar to that of CPV4 with the larger unit cell. Compared to wild-type CPV1, CPV1Δ crystals also had a slightly enlarged unit cell (103.9 *vs.* 103.0 Å). The absence of the base domain did not influence the overall structure of the trimeric building block but increased flexibility such that the electron density for 40–50 residues in the inter-trimer contact areas could not be observed. These disordered regions include the areas in CPV1 which had been shown previously to bind NTPs, so that NTPs are absent from CPV1Δ. The base domain is therefore a region that is neither required for proper folding of the protein, nor for crystal assembly, but fine-tunes the crystal, ‘locking-down’ the structure, often in conjunction with NTPs. This region is also possibly important for virion recognition and packaging, as discussed below. There are no contiguous channels in the wild-type polyhedra (Fig. S5), although there are significant voids where trimers come together to form dodecamers, and in the heart of the trimers. In CPV1, 14, 15, 17, 18 and 20 solvent molecules can move between these two voids, whereas in CPV4, 5 and 19 they are blocked by tyrosine residues. In contrast in the base domain deletion mutants all major cavities are connected, resulting in contiguous solvent channels running through the crystal lattice (Fig. S5).

### 3.6 Polyhedra act as molecular sieves modulating pH dependent disruption

**Fig. 3:**
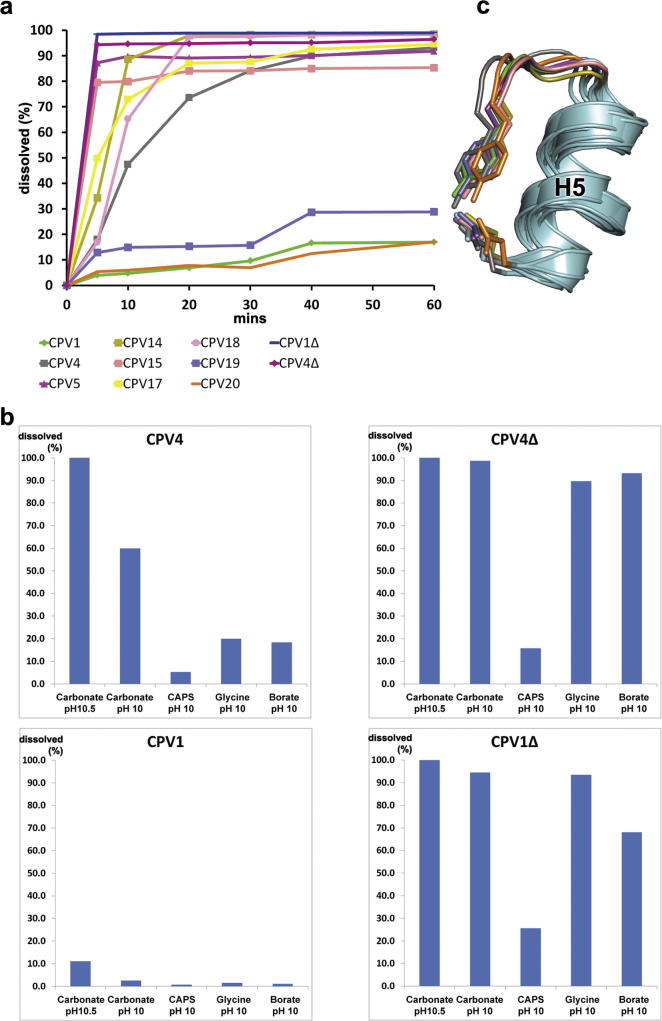
Polyhedra dissolution. (a) Time course of alkaline disruption of cypovirus polyhedra. The polyhedra of nine cypovirus types plus two deletion mutants were suspended in 50 mM carbonate buffer, pH 10.5 at room temperature and the non-dissolved material monitored by absorbance at 600 nm. This was converted to % disruption by comparison to the starting value. (b) Effect of five alkali pH buffers on polyhedra of 4 cypovirus types. Release of protein from insoluble polyhedra was monitored by A280 after 20 min. As a reference, aliquots of polyhedra were completely dissolved in carbonate buffer pH 12.0, and this was used to convert to % disruption. (c) Tyr-glu interaction conserved in 8 CPV types. The green star in Fig. 1a helps locate this panel to the overall structure (the views are identical). Colours of CPV types as in Fig. 1a.

One characteristic feature of polyhedra is that disruption only occurs when they are exposed to the very alkaline pH environment found in insect midguts, which is physiologically important to ensure the delivery of virus particles to the target intestinal cells (Rohrmann, 1986). To investigate how this critical process is achieved, eleven different cypovirus polyhedra (nine wild-type and two deletion mutants) were incubated in carbonate at pH 10.5 at room temperature for an hour, and the rate of disruption monitored by measurement of OD600 of the insoluble fraction (Fig. 3a). CPV1, CPV19 and CPV20 were resistant such that only a small portion was dissolved in an hour while the other six cypovirus types plus the two deletion mutants dissolved dramatically. To investigate if hindrance of access or buffer chemistry might be a factor in these differences we investigated a subset of polyhedra, CPV1, CPV4 and their deletion mutants, in a series of alkaline buffers at pH 10.0 for 30 min (Fig. 3b). Wild-type polyhedra behaved very differently in these buffers. Carbonate buffer is far more effective at dissolving polyhedra than other buffers, suggesting that the buffer structure as well as pH is important. The deletion mutants are much more sensitive to alkaline buffers, even those such as glycine and borate which do not disrupt crystals of full-length polyhedrin, although CAPS could not dissolve any of the samples effectively. Overall effectiveness is inversely correlated with size of the buffer molecule (Table S2) and the three CPVs (CPV1, 19 and 20) most resistant to alkali buffers have more extensive interactions between the threefold symmetry-related subunits at the CTP binding site, either strengthened by CTP (CPV1) and UTP (CPV20) binding (see below) or achieved by a large number of protein interactions mainly through hydrogen bonding, suggesting that physical access is a major factor in allowing dissolution, and that crystals tend to be resistant to non-physiological alkalis.

It has been proposed that a cluster of tyrosines trigger alkali disruption of both cypovirus (Coulibaly et al., 2007) and baculovirus (Chiu et al., 2012; Coulibaly et al., 2009; Ji et al., 2010) polyhedra, with some supporting mutagenesis data for CPV1 (Y. Ohtsuka et al., 2010). However, of the eight new structures, only CPV18 has a full tyrosine cluster of 8 residues as found in CPV1, the others have between 2 to 5. Nevertheless in all types except CPV17 the hydroxyl-group of conserved tyrosine 232 (CPV1/18 numbering) hydrogen bonds to another conserved residue, glutamate 217 (CPV1/18 numbering) (Figs. 3c and S2). We suggest that upon raising the pH, the tyrosine acquires a negative charge, repelling the glutamate, moving H5 and the C-terminal region and initiating disruption of the crystal lattice. This tyrosine is also vital for polyhedra assembly since mutating it ablates crystal formation (Ohtsuka et al., 2010).

### 3.7 NTPs and protein chains can exchange places whilst maintaining the same crystal lattice

In CPV1 three NTPs per polyhedrin subunit are specifically trapped within the protein matrix (Coulibaly et al., 2007), which might suggest that these nucleotides are essential for crystal formation and would be conserved during evolution. However, across our database of nine polyhedrin types we can now see that NTPs are dispensable (Table 2). CPV1 in fact bears the most NTPs and so we will relate our results to that type where the NTPs lie in two areas; ATP and GTP bind close to each other to form a purine cluster whilst CTPs amass at the centre of the dodecamer, the pyridine region.

#### 3.7.1 Purine cluster

**Fig. 4:**
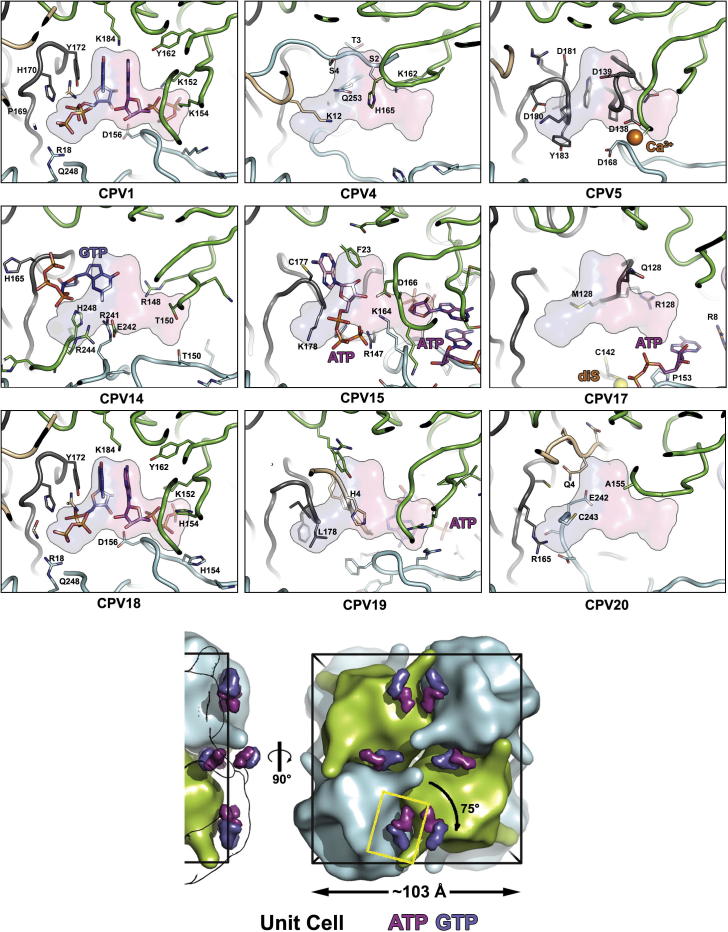
Purine binding. The bottom panel helps orient the reader to the position of the depicted in the other panels. Side and face-on views of a unit cell are shown, with smoothed maps representing polyhedrin trimers similar to Fig. 2. In the side view the 2 trimers closest to the viewer are shown only as outlines so that the close proximity of the NTPs to the surface of the unit cell can be clearly seen. To improve clarity the positions of the ATP (magenta) and GTP (blue) moieties for just CPV1 are shown in this panel. As a reference the positions of the CPV1 ATP and GTP have been replicated across the other panels. The yellow box shows the area represented in other 9 panels. For the nine cypovirus types the same viewpoint is shown. Protein chains are coloured separately and consistently between types. Side chains of residues which either interact with nucleotides or occupy nucleotide pockets are shown. The view has narrow clipping planes to facilitate interpretation. The positions of the calcium ion in CPV5 and C142 in CPV17 are indicated.

The purines are found at three 2-fold axes on the facets such that there are eight symmetry equivalent purine clusters per facet (Fig. 4). Each has complex interactions with the neighbouring protein, including variable regions V1, V3, V4, V2c and V5 contributed by four different polypeptide chains, forming diverse interactions between the facets of unit cells in different polyhedra (Fig. S6). In CPV18 purine binding is almost identical to CPV1 in that GTP hydrogen bonds with V2c and V1 while ATP is supported by two V4 regions from different polypeptide chains. The ATP and GTP bases stack with each other and with the phenol ring of Y172. There are two substitutions in the binding pocket, K154H and R155V. Residue 155 interacts via the main chain nitrogen, and is thus sequence independent, whilst at residue 154 both the lysine and histidine side chain NH groups hydrogen bond with ATP phosphates.

CPV4, 5 and 20 lack both ATP and GTP, whilst the other cypovirus types have varying combinations, in all cases the V1, V3, V4, V2c or V5 regions of the protein adopt alternate polypeptide conformations (Fig. 4). Thus in CPV4, the purines are replaced by greatly elongated V1 and V5 regions, whilst the shortened V4 loop would no longer be able to contact the ATP. In CPV20 the V4 loop is similarly shortened whilst V1, V2c, and V5 infiltrate the region. In CPV5 V3 (179–187) and V2c (135–140) form clamp-like protrusions which fill the GTP and ATP pockets, whilst in the SeMet form a calcium ion links four polyhedrin molecules (Fig. S3).

CPV15 is similar to CPV5 however V3 and V2c are shorter, leaving space for two ATPs in different conformations to CPV1: one binds V3 and V2c and the other stacks at the 2-fold axis with the backbone of V4 forming a further layer. In CPV14, a GTP is located ∼5 Å from the CPV1 position, in a different conformation, binding to the backbone of V2c and embraced by V1 and bulky side chains of an elongated V5. A change in the conformation of the V4 hairpin switches it to bind another monomer. In CPV19, an extra V2c loop and elongated V1 eliminates the GTP space completely and displaces the ATP base ∼13 Å *cf* CPV1, to a site surrounded by V4 and V5.

In summary the two purines are shrouded by amino acids, leaving almost no cavity (Fig. 4) and bridge at least four protein molecules to reinforce interactions between adjacent unit cells. Where one or both are missing, the polypeptide is altered to substitute for the missing moieties.

#### 3.7.2 Pyridine region

**Fig. 5:**
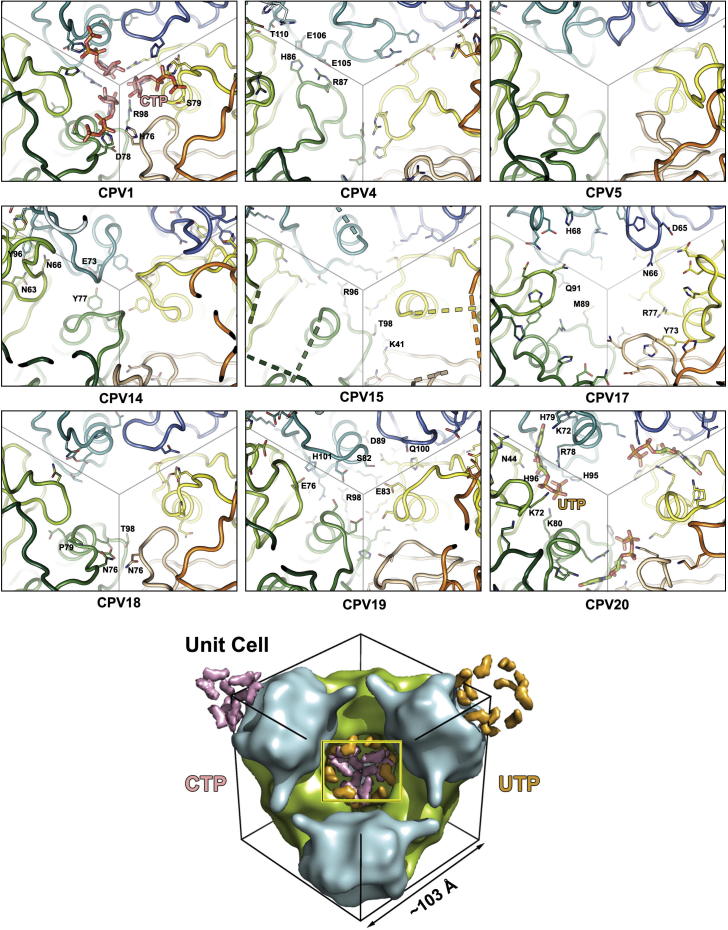
Pyrimidine binding. The lower panel orientates the reader to the positions of the CTPs and UTPs within the unit cell which cluster around the centre and the apexes. The yellow box shows the area represented in other 9 panels. The view for the nine cypovirus types is from the centre out along a body diagonal. Each trimer is roughly denoted by lines. The 9 subunit protein chains visible in the view are coloured separately with the shading grouped according to which trimer they belong to (blue, green and red-yellow). Nucleotide moieties are shown as sticks along with side chains important in this region. The 13 residues missing from the CPV15 model are shown by dashed lines.

NTP binding in this area is only observed in CPV1 and CPV20. In CPV1 a ball of 12 CTPs lie at the point of *23* symmetry at the heart of the dodecamer (Fig. 5). The pyridine in CPV20 is not well ordered, but can be identified as UTP based on the limited space and the hydrogen bonding environment provided by residue 101 main chain nitrogen and the side chain nitrogen of H79, whilst the triphosphates are supported by two lysines, two histidines and an arginine. The pyridines stabilise the dodecamer, although in CPV1 the CTPs also interact with each other, whilst the UTPs in CPV20 are more deeply buried in the trimer interface (Figs. 5 and S7).

In the cypovirus types which contain no pyridine the proteins take up a number of different configurations. In CPV14 and CPV19 the V2n loops move to occupy some of the central space. CPV4 has limited numbers of molecular interactions in this area, while in CPV5 a few strong trimer-trimer interactions are present. In CPV15, 13 disordered residues are situated around this region (indicated by dashed lines in Fig. 5) leaving the trimer contacts apparently weakened. Despite a strong overall similarity with CPV1 there is no pyridine bound in CPV18. Many of the key residues that bind the CTP in CPV1 (H76, N77, D78, S79, Y80, D81, D96 and R98) are substituted, including H76N and S79P, which interact with the phosphates and R98T which stacks with the base. Clearly these changes are sufficient to disrupt CTP binding (Fig. 5).

To summarise, different polyhedrins adopt different solutions to stabilising the crystal lattice, some use specific nucleotides, whilst others substitute protein chains, in all crystals however the lattice is conserved.

## 4 Discussion

**Fig. 6:**
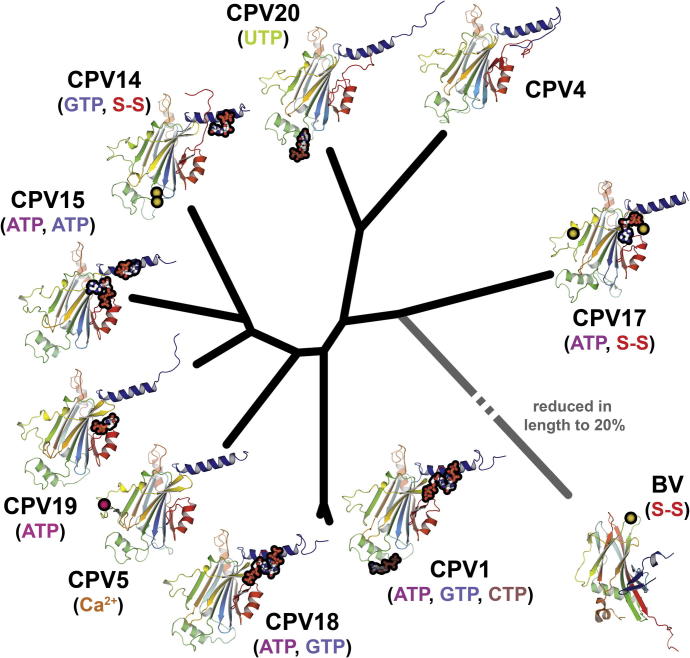
Phylogenetic tree showing relationship between the polyhedrin proteins. Superpositions were performed using SHP (Stuart et al., 1979) and the phylogenetic tree calculated with PHYLIP (Felsenstein, 1989). The length of the branch to baculovirus polyhedrin (BV) has been reduced to 20% of its actual length. Structures of the proteins are shown coloured from N-terminus to C-terminus, blue to red. NTPs are represented in thickened stick style with a black outline to aid visualization. Cysteines involved in disulphide bridges are shown as black spheres, and the calcium ion in CPV5 as a pink sphere.

Structures for a number of previously uncharacterised types of cypovirus polyhedrins have revealed a strongly conserved structural core. A phylogenetic tree constructed based on the structures (Fig. 6) shows, as expected, that CPV1 and CPV18 are closely related structurally, but otherwise the tree is rather star-like, with CPV17 being perhaps rather an outlier and closest to the markedly different baculovirus polyhedrin. It is conceivable that CPV17 retains some features of an ancestral insect virus polyhedrin. Given the evolutionary explosion of insects some 400 million years ago the considerable divergence of the polyhedra suggests that much of the substantial evolution of the CPVs took place alongside these events, perhaps explaining why the build-up of variation appears almost saturated, with only two amino acids fully conserved across all nine types. Away from the core of the polyhedrin we identify five regions of structural variability and show that NTPs are used in the assembly of some but not all proteins, so that they impose little constraint on the polyhedrin structure. We also find that the whole base domain is dispensable for polyhedra formation, although the biochemical features are altered dramatically when it is removed. The base domains include the most variable region of the protein and nestle around a point of *23* symmetry. The bases likely attach to the virus to be encapsidated and are therefore either packed together inside the crystal or exposed on the outside, furthermore, since they do not drive assembly they will tend to remain accessible on the surface of the growing crystal, ready to engage a protein-only icosahedral shaped CPV particle. The outer faces of the base domains are positively charged across nearly all the types, at present there is no little structural information for charge status of the virion component that is likely to attach to the polyhedrin. It seems that in the final stage of assembly the base domains pull the lattice into a closer knit form, presumably allowing useful flexibility in the lattice in the vicinity of the viral inclusions (note that the presumed ancestral CPV17 does not fully lock down the lattice, retaining a larger unit cell). The variety of NTPs seem to act as small molecule ‘fillers’ in the absence of virus particles, allowing lattice completion and the avoidance of crystal voids whilst permitting appropriate specificity in the virus recognition motifs between CPV types. Overall this domain is likely a late addition to the fold (it is dispensable for protein folding) and probably was a feature acquired to aid viral recognition (it is distal to the point of attack by ions which initiate the dissolution process by modifying a point critical for assembly). There may be potential for exploiting the variation in the base domain for nanotechnology applications.

Structure based sequence alignments of the polyhedrins show that whilst approximately 80% of the C alphas are structurally equivalent (pairwise rmsd from 0.2 to 1.5 Å), the corresponding sequence identities are only 12–32% (Fig. S2). The exception is the polyhedrin of CPV18, which has >80% sequence identity with that of CPV1, consistent with their relatively recent divergence (Graham et al., 2007). Although the two molecules are very similar (rmsd of 247 structurally equivalent Cαs 0.23 Å (Fig. S1)) they behave dramatically differently in alkali buffers, CPV18 being far more sensitive to dissolution. Clearly functional differences have been locked into these molecules, probably arising from the structure around the CTP binding site. In fact the three CPVs (CPV1, 19 and 20) most resistant to alkali buffers all display more extensive interactions between the 3-fold symmetry-related subunits of the trimeric building block – strengthened by either pyridine binding (CPV1 and CPV20) or numerous protein–protein hydrogen bonds.

Overall polyhedra present a fascinating picture of the effect of 400 million years of evolution on a system where crystal lattice formation is conserved in the face of massive sequence variability. By spreading the crystal contacts over the majority of the surface of the molecule lattice formation remains robust in the face of many individual changes, in the same way that the tertiary structure is robust. Indeed whilst most proteins harbour the greatest fraction of conserved residues in the protein core (e.g. 63% for picornavirus 3C proteases) only a minority (43%) of the conserved residues in CPV polyhedrins are buried in the core.

## Accession numbers

Coordinates and structures factors have been deposited in the PDB with accession codes: CPV1Δ 5A9B; CPV4 101 Å cell 5A8S; CPV4 104 Å cell 5A8T; CPV4Δ 5A9C; CPV5 native 5A8U; CPV5 SeMet 5A8V; CPV14 5A96; CPV15 5A98; CPV17 room temperature 4S1L; CPV17 100K 4S1K; CPV18 5A9P; CPV19 5A99; CPV20 5A9A.

## Figures and Tables

**Table 1:** Data collection.

	CPV4 Cell 1	CPV4 Cell 2	CPV5	CPV5 SeMet	CPV14	CPV14 SeMet	CPV15	CPV15 SeMet	CPV17 room temp	CPV17 frozen	CPV18	CPV19	CPV19 SeMet	CPV20	CPV1Δ	CPV4Δ
Number of crystals	18	11	26	128	20	13	40	112	5787	768	21	34	61	51	13	21
Crystal size (μm)	5–7	5–7	3–4	3–4	3–4	3–4	∼1.5	∼1.5	∼1.0	∼1.0	4–5	∼3	∼3	3–4	4–6	5–7
Cell *a* = *b* = *c* (Å)	101.7	104.8	102.3	102.1	102.6	102.8	102.9	103.5	106.1	104.9	102.8	102.7	103.3	103.4	103.9	104.3
Resolution (Å)^a^	29.4–1.72 (1.82–1.72)	28.0–2.00 (2.11–2.00)	51.2–1.61 (1.65–1.61)	72.2–2.07 (2.14–2.07)	72.5–1.91 (2.02–1.91)	36.3–2.24 (2.36–2.24)	51.4–1.82 (1.86–1.82)	51.7–2.48 (2.55–2.48)	28.3–1.75 (1.79–1.75)	74.16–2.20 (2.26–2.20)	72.7–1.48 (1.51–1.48)	51.4–1.50 (1.59–1.50)	42.1–2.03 (2.08–2.03)	51.7–1.82 (1.87–1.82)	42.4–1.88 (1.93–1.88)	33.0–1.71 (1.75–1.71)
Unique reflections	17233 (1580)	11543 (797)	23283 (1726)	10902 (1093)	12952 (1045)	7956 (632)	16418 (1193)	6666 (473)	19096 (1032)	9376 (931)	30415 (2238)	21653 (498)	11977 (870)	16582 (1219)	15023 (1093)	19985 (1319)
*R*_merge_	0.199 (0.385)	0.177 (0.330)	0.342 (0.000)⁎⁎	0.163⁎ (0.387⁎)	0.199 (0.327)	0.186 (0.363)	0.468 (0.000)⁎⁎	0.499 (0.000)⁎⁎		0.665 (0.000)	0.320 (0.000)⁎⁎	0.368 (0.000)⁎⁎	0.437 (0.000)⁎⁎	0.342 (0.000)⁎⁎	0.226 (0.000)⁎⁎	0.189 (0.000)⁎⁎
*I/*σ*I*	9.7 (1.7)	8.1 (1.7)	7.0 (1.4)	7.5 (1.9)	8.4 (1.8)	7.1 (1.6)	4.2 (0.8)	6.4 (1.8)		6.4 (1.4)	7.1 (1.5)	3.8 (0.3)	5.4 (1.0)	5.4 (1.1)	4.8 (1.0)	5.0 (0.6)
Completeness (%)	92.7 (59.0)	88.5 (42.7)	100 (100)	100 (100)	92.3 (52.1)	90.0 (50.3)	99.7 (97.9)	100 (100)	100 (100)	99.9 (100)	100 (99.8)	75.5 (12.2)	98.7 (91.5)	100 (99.7)	98.8 (98.6)	97.3 (88.9)
Redundancy	11.4 (1.9)	7.6 (1.5)	14.8 (10.7)	64.1 (26.1)	7.9 (1.6)	6.7 (1.5)	7.8 (4.5)	19.8 (13.3)	52.4 (11.1)	47.8 (25.4)	12.9 (8.6)	3.8 (1.1)	14.3 (6.4)	10.0 (6.5)	4.3 (4.0)	4.4 (2.7)

CPV17 from (Ginn et al., 2015b).

a Values in parentheses are for the highest resolution shell.

⁎ *R*_merge_ obtained by scaling of two already processed data sets.

⁎⁎ *R*_merge_> = 1.

**Table 2:** Refinement statistics.

	CPV4 Cell 1	CPV4 Cell 2	CPV5	CPV5 SeMet	CPV14	CPV15	CPV17 room temp	CPV17 frozen	CPV18	CPV19	CPV20	CPV1Δ	CPV4Δ
Resolution (Å)	29.4–1.72	28.0–2.00	51.2–1.61	36.1–2.07	32.4–1.91	42.0–1.82	28.30–1.75	74.16–2.20	72.7–1.48	41.9–1.51	51.7–1.83	42.4–1.88	27.9–1.71
No. of reflections	17233	11542	23282	10889	11856	16408	20122	9376	30414	20976	16323	15016	19908
*R*_work_/*R*_free_	0.118/0.166	0.175/0.239	0.159/0.197	0.169/0.228	0.141/0.184	0.162/0.220	0.122/0.154	0.147/0.199	0.130/0.148	0.166/0.228	0.157/0.190	0.189/0.251	0.185/0.216
No. of atoms													
Protein	2028	1637	2002	2002	2022	1952	1914	1907	2001	2058	1979	1686	1628
Ligand (#NTPs)	0	0	0	1 (0)	32 (1)	89 (2)	32 (1)	32 (1)	63 (2)	32 (1)	29 (1)	0	0
Water	176	92	166	112	165	176	174	146	246	235	161	101	136
B-factors													
Protein	13.2	22.8	12.6	14.2	12.7	16.7	23.7	22.5	8.4	14.3	21.4	36.0	26.9
Ligand	–	–	–	51.0	49.8	28.4	34.4	50.0	31.0	18.5	47.1	–	–
Water	20.1	28.4	19.6	16.3	17.3	21.8	32.7	32.2	16.8	20.8	25.6	36.1	37.0
rmsd													
Bond lengths (Å)	0.006	0.006	0.008	0.006	0.006	0.007	0.010	0.013	0.005	0.007	0.003	0.003	0.003
Bond angles (°)	1.09	0.98	1.19	1.02	1.08	1.28	1.39	1.675	1.146	1.12	0.83	0.79	0.74
rmsZ													
Bond length	0.39	0.34	0.41	0.32	0.40	0.37	0.50	0.68	0.30	0.38	0.23	0.26	0.23
Bond angles	0.56	0.52	0.58	0.49	0.57	0.53	0.63	0.82	0.52	0.53	0.43	0.44	0.42
Model quality (Ramachandran plot)													
Most favoured (%)	98.8	98.0	98.0	97.6	96.0	96.6	97.9	97.4	97.6	97.6	97.9	97.1	98.0
Allowed (%)	100.0	100.0	100.0	100.0	100.0	100.0	100.0	100.0	100.0	100.0	100.0	100.0	100.0
PDB ID code	5A8S	5A8T	5A8U	5A8V	5A96	5A98	4S1L	4S1K	5A9P	5A99	5A9A	5A9B	5A9C

## Appendix A Supplementary data

Supplementary data This document contains Supplementary Figs. S1–S7 and Tables S1, S2.
