# Supplementary material for: Polyhedra structures and the evolution of the insect viruses
Source: J Struct Biol. 2015 Oct;192(1):88–99. doi: 10.1016/j.jsb.2015.08.009 (PMC4597613; doi:10.1016/j.jsb.2015.08.009)
Supplement: Supplementary data — This document contains Supplementary Figs. S1–S7 and Tables S1, S2. [file mmc1.docx]

**Table S1.** List of cypovirus polyhedrins studied.

| Type | Serotype | GeneBank Code mRNA | Origin | Host Order | Geographical location of insect isolate | Abbreviation used |
| --- | --- | --- | --- | --- | --- | --- |
| 1 | BmCPV1 | AB003361 | Bombyx mori | Lepidoptera | Japan | CPV1 |
| 4 | AmCPV4 | AY212272 | Antheraea mylitta | Lepidoptera | India | CPV4 |
| 5 | OpCPV5 | U06196 | Orgyia pseudotsugata | Lepidoptera | USA, pacific northwest | CPV5 |
| 14 | LdCPV14 | AF389461 | Lymantria dispar | Lepidoptera | China | CPV14 |
| 15 | TnCPV15 | AF291692 | Trichoplusia ni | Lepidoptera | Not specified. T.ni is found worldwide | CPV15 |
| 17 | UsCPV17 | AY876384 | Uranotaenia sapphirina | Diptera | Florida, USA | CPV17 |
| 18 | ObCPV18 | DQ192250 | Operophtera brumata | Lepidoptera | Orkney, UK | CPV18 |
| 19 | ObCPV19 | DQ192254 | Operophtera brumata | Lepidoptera | Orkney, UK | CPV19 |
| 20 | SuCPV20 | DQ834386 | Simulium ubiquitum | Diptera | Florida,USA | CPV20 |

**Table S2.** The active species of the alkaline buffers used in the solubility study of polyhedra.

| **Buffer** | **Major active**  **species** | **Structure formula** | **Size of alkaline buffer effective species** |
| --- | --- | --- | --- |
| Carbonate | CO_3_^2^ˉ | 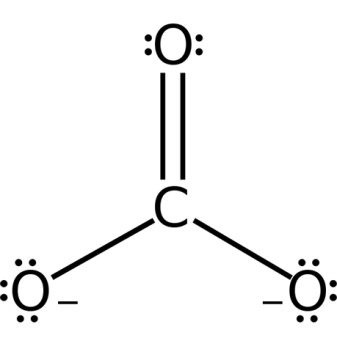 | 46.842 |
| Borate | B(OH)_4_ˉ | 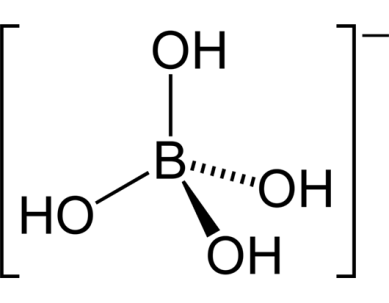 | 58.590 |
| CAPS | C_9_H_19_NO_3_S | 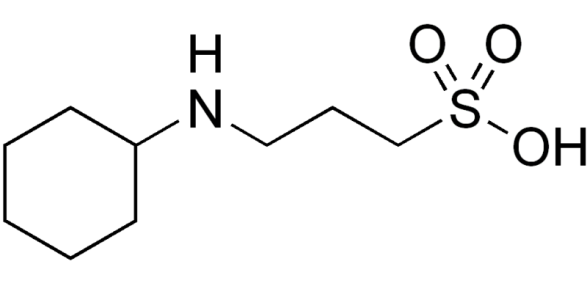 | 176.097 |
| Glycine | Glycinate anion | 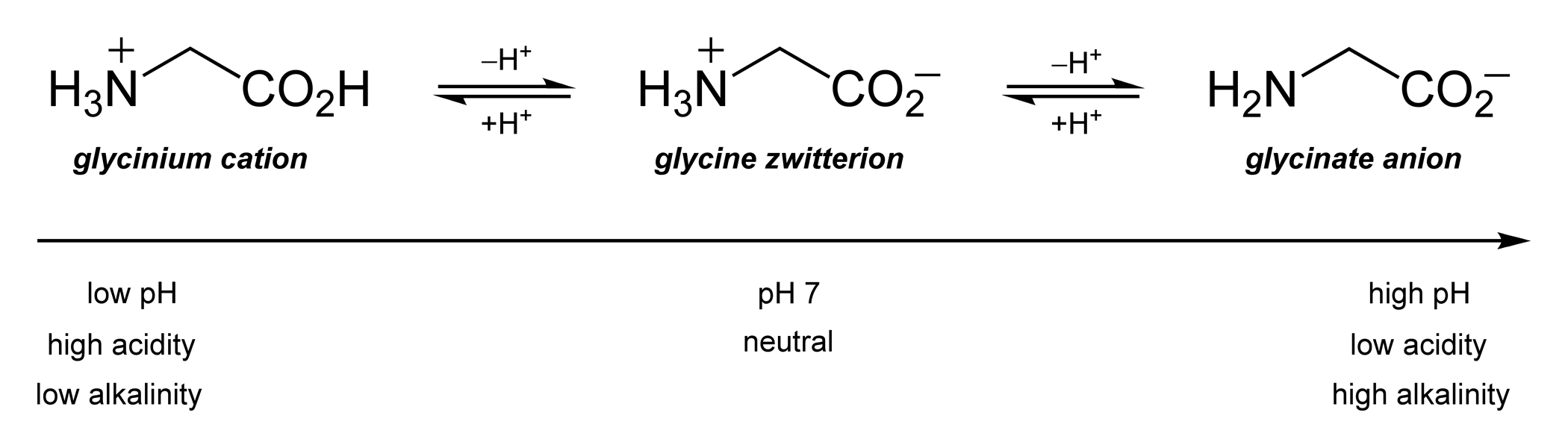 | 64.109 |


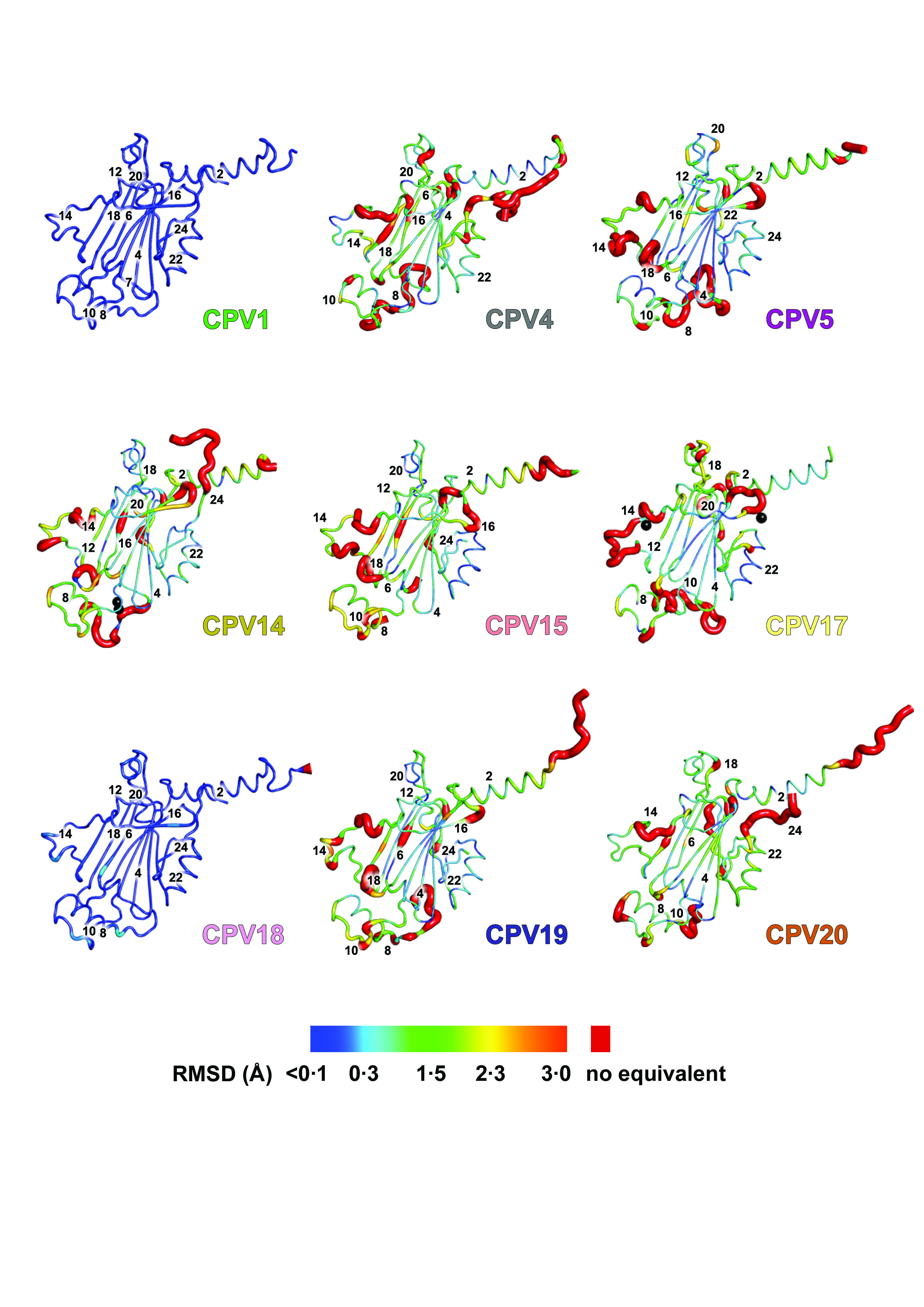


**Fig.S1.** Comparison of the cypovirus polyhedrin structures. The molecules have been aligned with SHP using CPV1 as the reference. The colour spectrum (blue to orange) and cartoon tube thickness represent root mean square (rms) distance of the c-alphas from the CPV1 structure. Regions which have no alignment are coloured red and displayed with exaggerated thickness. Cysteines involved in disulphide bond formation in CPV14 and CPV17 are marked by black spheres. Structures are numbered every 20^th^ residue where possible; note that the trailing zero is omitted to avoid clutter, so 18 marks residue 180 for instance.


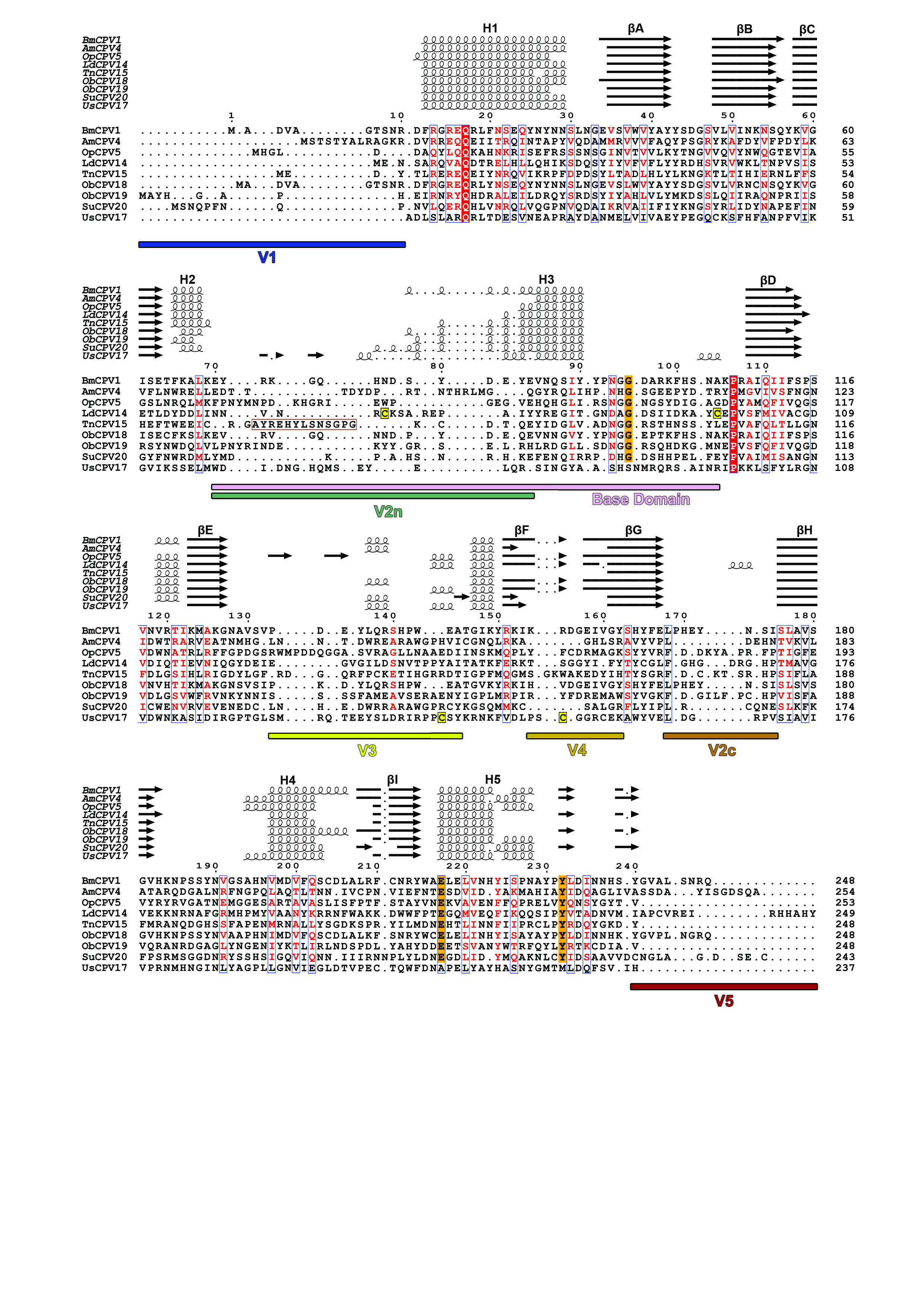


**Fig.S2.** Structure based sequence alignment. Sequence alignment based on 3-D structure superposition. Secondary structures are marked above the sequences and are labelled as in Fig. 1a. Fully conserved residues are marked by a solid red box with white character, similar residues with a red character. Similarity is calculated according to the Risler matrix using the program ESPript ([Robert and Gouet, 2014](#_ENREF_29)). Residues conserved across 8 CPV types (excludes CPV17) are boxed in orange. Variable regions V1-V5 are shown and the base domain is coloured oxley. The thirteen residues of CPV15 which could not be built into the model are boxed in maroon. Cysteines involved in the CPV14 intra-chain disulphide and CPV 17 inter-chain disulphide are boxed in yellow. Amino acid numbering at the top is for CPV1 with residue numbers for all types along the right side.


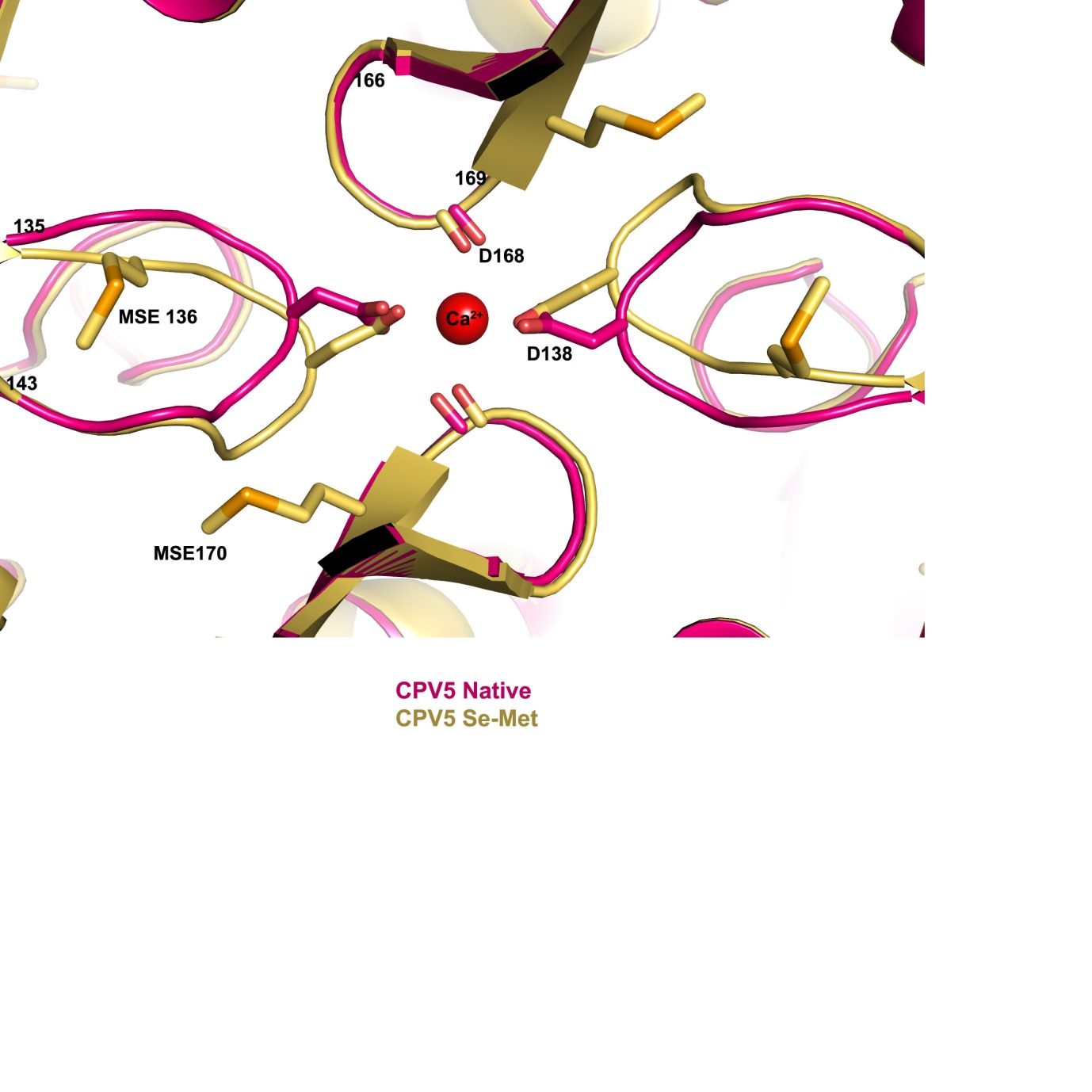


**Fig.S3.** CPV5 Ca^2+^. Detail of the Ca^2+^ ion co-ordination in CPV5 SeMet structure and comparison with the native protein.


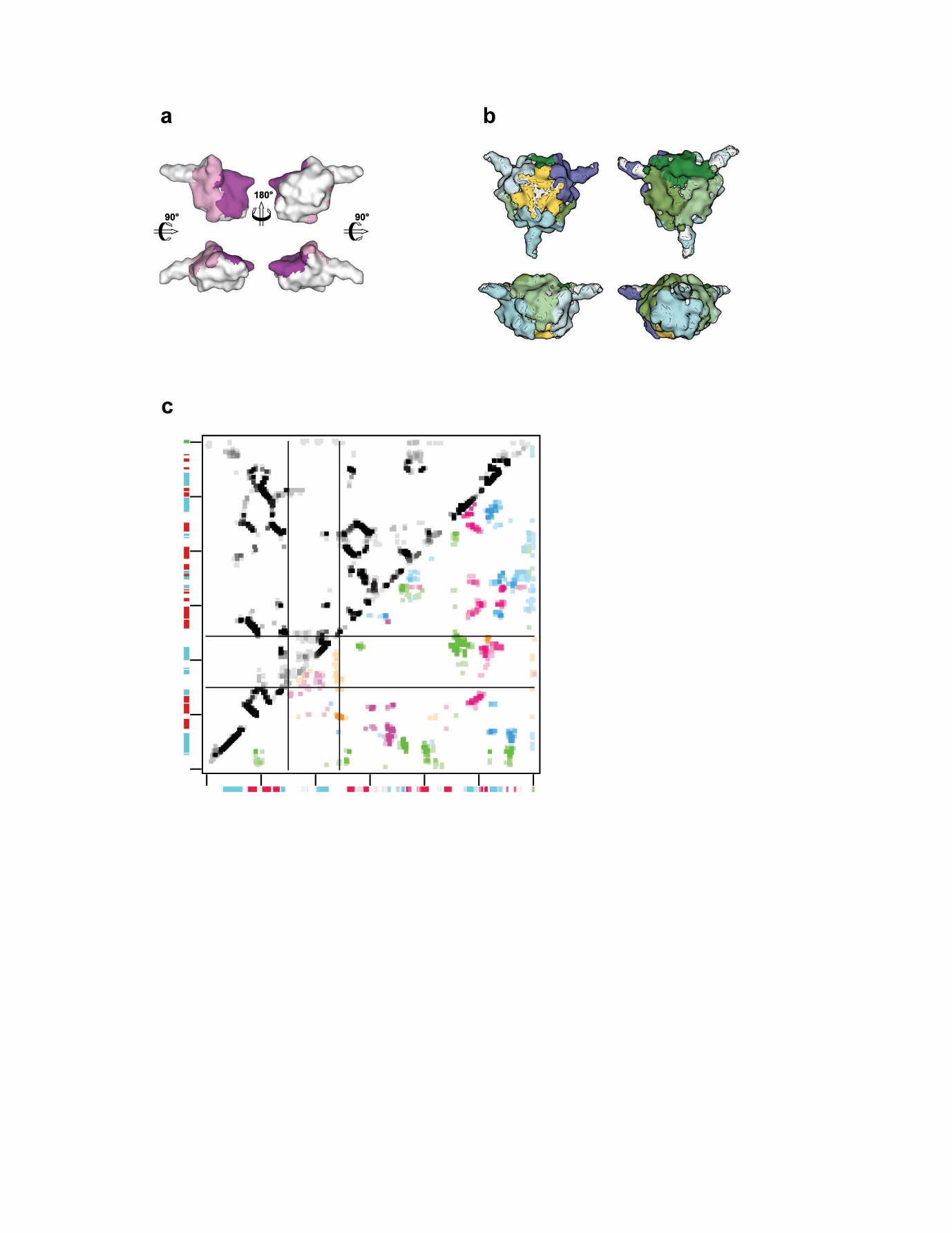


**Fig.S4.** Interface regions mapped onto monomer and trimer. (a) Surface representations of a monomer showing the contacts made by 2 neighbouring molecules forming a trimer, coloured pink and magenta. (b) Surface representations of a trimer showing the areas principally involved in inter-trimer contacts. Trimer-trimer contacts are in shades of green, dodecamer formation is shown in blue and trimer-trimer contacts involving the base domain in yellow. (c) Representation of the intra- (top left half of graph) and inter-molecular (bottom right) contacts for the residues of the nine polyhedrin molecules. Residues involved in contacts were determined by the program CONTACTS (R.Esnouf, unpublished program). Contacting residues within 2 of the target were ignored for clarity. Intermolecular contacts are coloured according to which molecule is making the contact in the lattice, similar to panels (a) and (b) such that monomer-monomer are coloured pink/magenta, trimer-trimer are green, dodecamer formation is shown in blue and trimer-trimer contacts involving the base domain in yellow. The saturation of the colour indicates how many of the nine polyhedrins contribute to a particular contact; the more saturated the colour the greater number of polyhedrins that have that contact. Intramolecular contacts are in shades of gray and as above the darker the colour the more polyhedrins contribute to that particular contact. The secondary structure is shown along the axes, cyan box for α-helix and red for β-sheet with the saturation of the colour denoting the number of types of polyhedra for which this contact was observed. The intersection of the 4 lines delimits is the base domain. The axes have subdivisions every 50 amino acids.

**
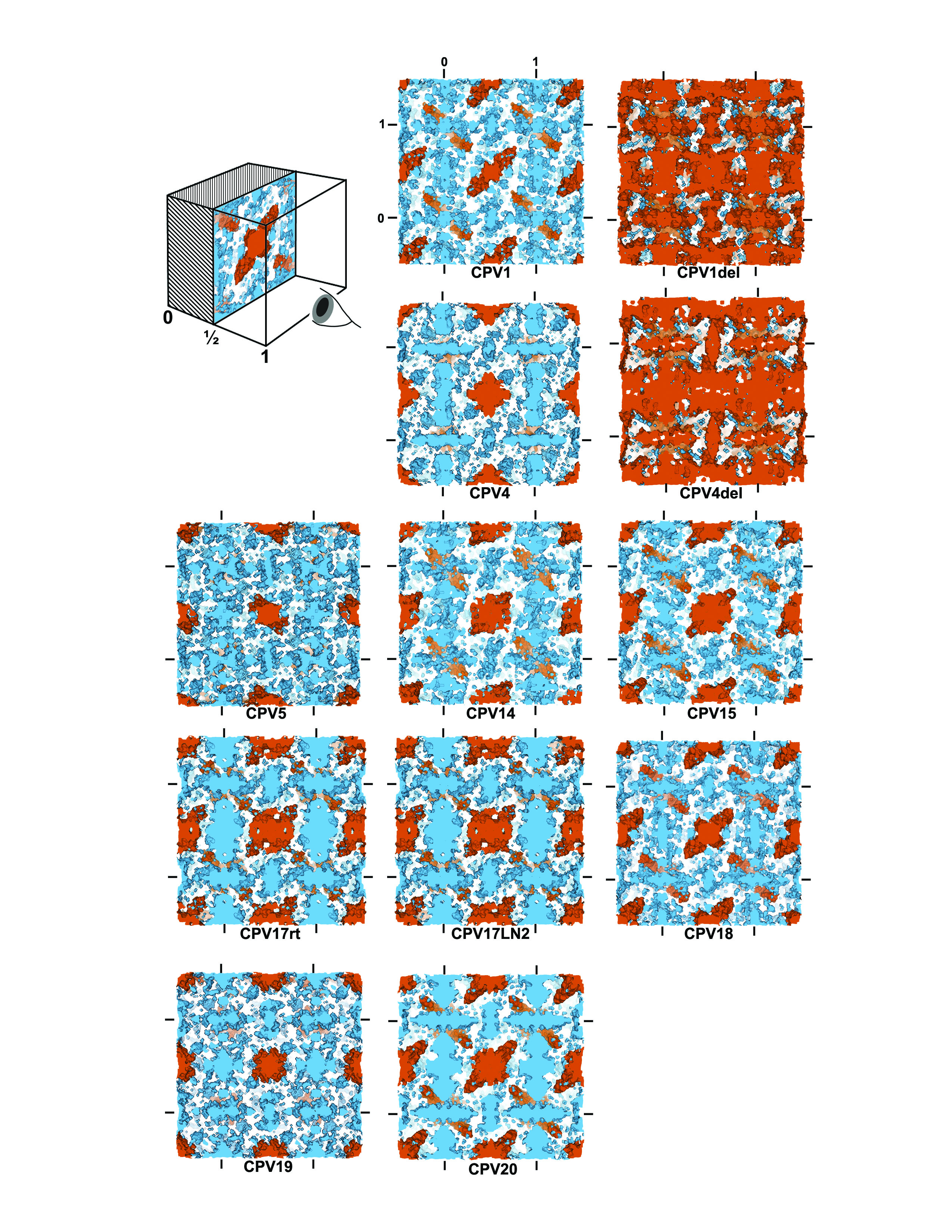
 Fig.S5.** Solvent cavities in the crystal. The solvent accessible areas are coloured, with those areas which connect to the centre of the unit cell (and symmetry related equivalents) in vermillion and other cavities in sky blue. Each panel is two unit cell dimensions in length (approx. 200Å) centred on a ½, ½, ½ position in the cell and the view is clipped from a plane ½ along the cell, as depicted in the top left panel. For each panel the unit cell origin (0) and extent (1) are indicated by markers.


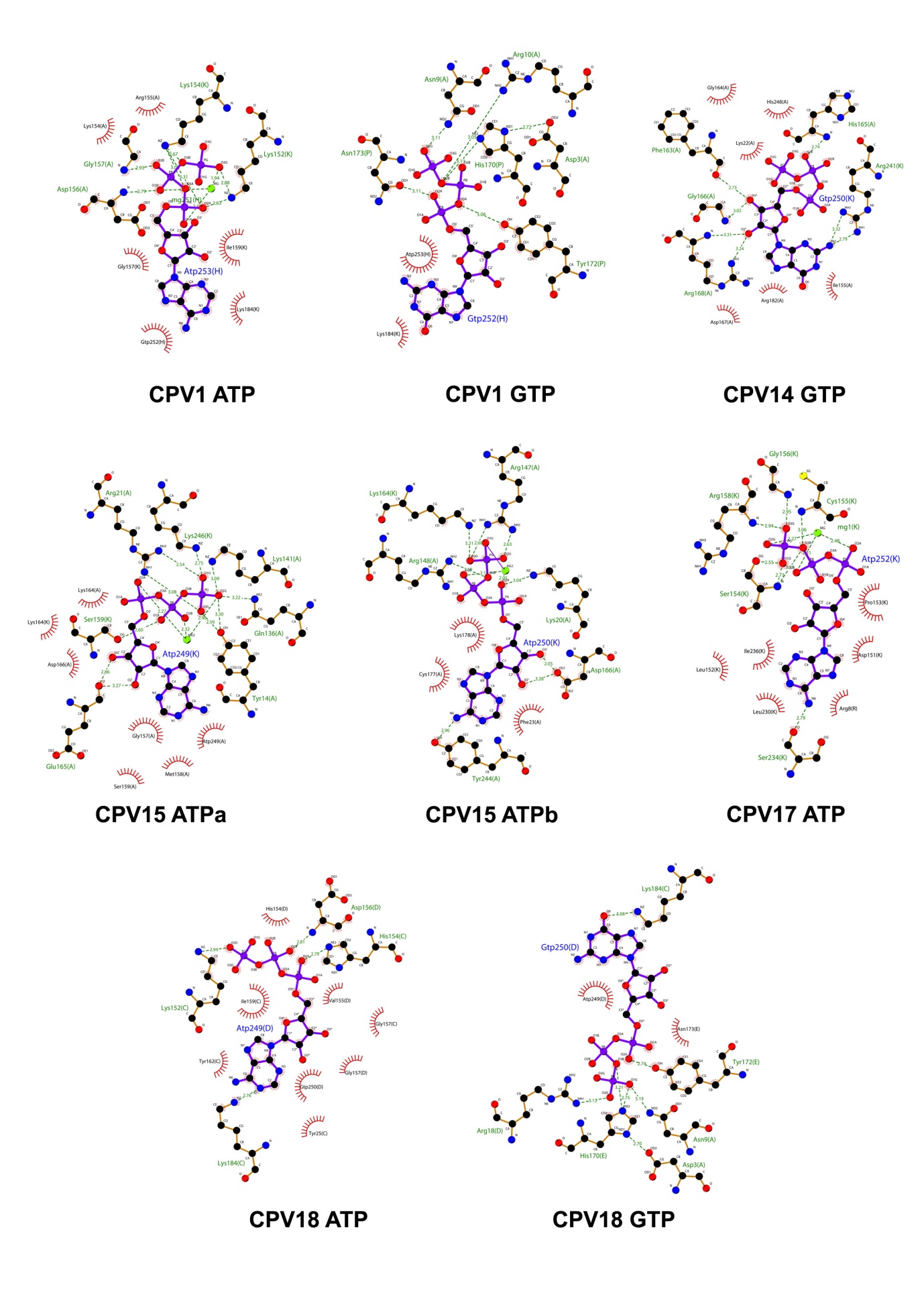


**Fig.S6.** LigPlot analysis of purine interactions.


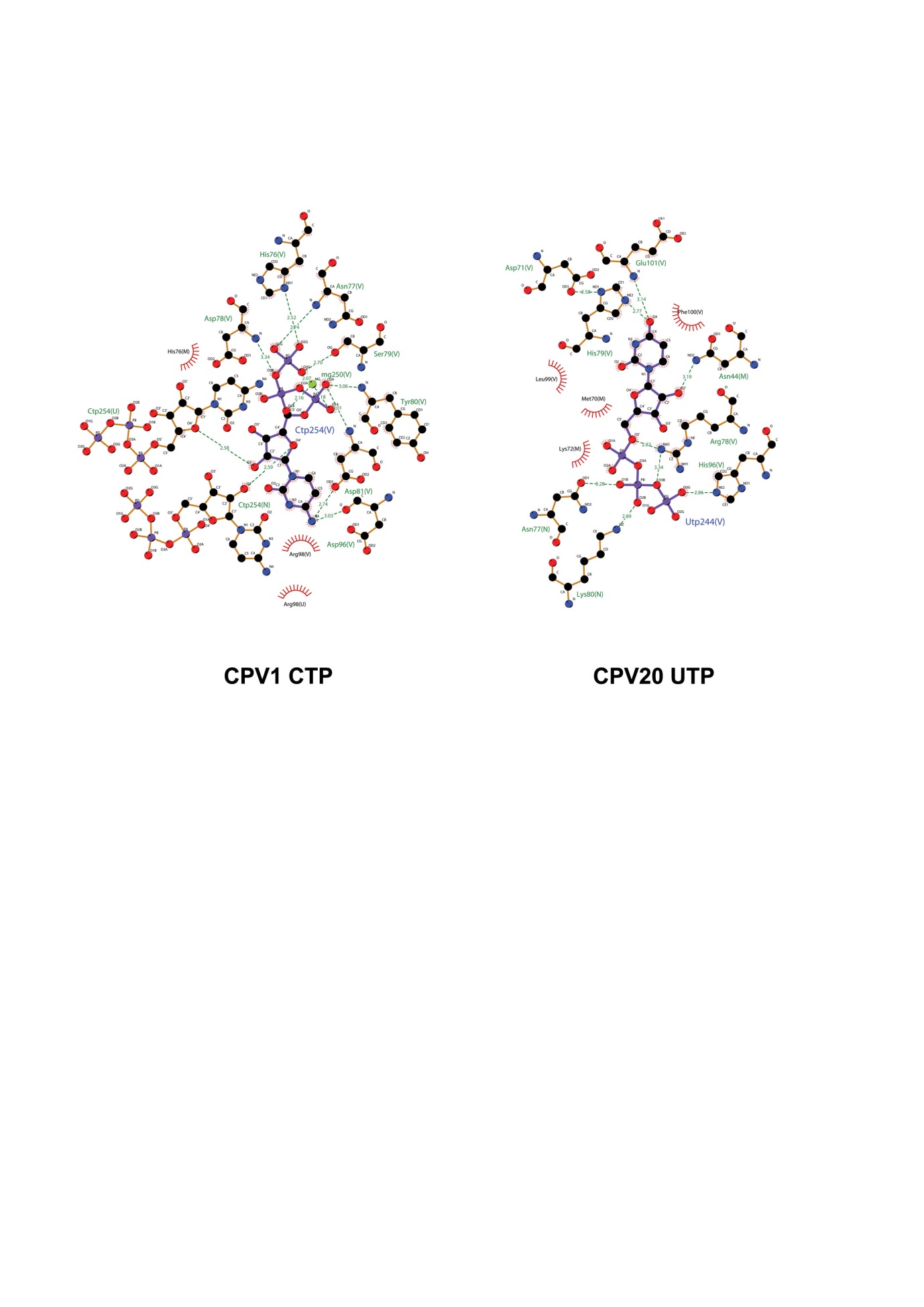


**Fig.S7.** LigPlot analysis of pyrimidine interactions.
